# Biomechanical Effects of the Porous Structure of Gyroid and Voronoi Hip Implants: A Finite Element Analysis Using an Experimentally Validated Model

**DOI:** 10.3390/ma16093298

**Published:** 2023-04-22

**Authors:** Zatul Faqihah Mohd Salaha, Muhammad Imam Ammarullah, Nik Nur Ain Azrin Abdullah, Aishah Umairah Abd Aziz, Hong-Seng Gan, Abdul Halim Abdullah, Mohammed Rafiq Abdul Kadir, Muhammad Hanif Ramlee

**Affiliations:** 1 Bone Biomechanics Laboratory (BBL), Department of Biomedical Engineering and Health Sciences, Faculty of Electrical Engineering, Universiti Teknologi Malaysia, Johor Bahru 81310, Johor, Malaysia; 2 Bioinspired Devices and Tissue Engineering (BIOINSPIRA) Research Group, Universiti Teknologi Malaysia, Johor Bahru 81310, Johor, Malaysia; 3 Department of Mechanical Engineering, Faculty of Engineering, Universitas Pasundan, Bandung 40153, West Java, Indonesia; 4 Biomechanics and Biomedics Engineering Research Centre, Universitas Pasundan, Bandung 40153, West Java, Indonesia; 5 Undip Biomechanics Engineering & Research Centre (UBM-ERC), Universitas Diponegoro, Semarang 50275, Central Java, Indonesia; 6 School of AI and Advanced Computing, XJTLU Entrepreneur College (Taicang), Xi’an Jiaotong-Liverpool University, Suzhou 215400, China; 7 School of Mechanical Engineering, College of Engineering, Universiti Teknologi MARA, Shah Alam 40450, Selangor, Malaysia; 8 Medical Devices and Technology Centre (MEDiTEC), Institute of Human Centered Engineering (iHumEn), Universiti Teknologi Malaysia, Johor Bahru 81310, Johor, Malaysia

**Keywords:** finite element, hip implant, lattice structure, Gyroid, Voronoi

## Abstract

Total hip arthroplasty (THA) is most likely one of the most successful surgical procedures in medicine. It is estimated that three in four patients live beyond the first post-operative year, so appropriate surgery is needed to alleviate an otherwise long-standing suboptimal functional level. However, research has shown that during a complete THA procedure, a solid hip implant inserted in the femur can damage the main arterial supply of the cortex and damage the medullary space, leading to cortical bone resorption. Therefore, this study aimed to design a porous hip implant with a focus on providing more space for better osteointegration, improving the medullary revascularisation and blood circulation of patients. Based on a review of the literature, a lightweight implant design was developed by applying topology optimisation and changing the materials of the implant. Gyroid and Voronoi lattice structures and a solid hip implant (as a control) were designed. In total, three designs of hip implants were constructed by using SolidWorks and nTopology software version 2.31. Point loads were applied at the x, y and z-axis to imitate the stance phase condition. The forces represented were x = 320 N, y = −170 N, and z = −2850 N. The materials that were used in this study were titanium alloys. All of the designs were then simulated by using Marc Mentat software version 2020 (MSC Software Corporation, Munich, Germany) via a finite element method. Analysis of the study on topology optimisation demonstrated that the Voronoi lattice structure yielded the lowest von Mises stress and displacement values, at 313.96 MPa and 1.50 mm, respectively, with titanium alloys as the materials. The results also indicate that porous hip implants have the potential to be implemented for hip implant replacement, whereby the mechanical integrity is still preserved. This result will not only help orthopaedic surgeons to justify the design choices, but could also provide new insights for future studies in biomechanics.

## 1. Introduction

According to the Food and Drug Administration (FDA), hip implants are classified as one of the medical devices which help to restore mobility and contribute to reducing pain in patients who are commonly associated with arthritis, hip diseases and injuries [1]. Each design for a hip implant has its own unique identity, such as shape [2], material [3], design [4], and dimensions [5]. The same hip implant systems can produce different outcomes in different patients. In a situation when the joint is destroyed or damaged, artificial joint replacement of the hip, called a hip implant, can be a substitute to the normal hip, providing excellent results [6]. However, during a complete total hip arthroplasty (THA) procedure, the hip implant inserted in the femur will not only kill the supply of the main artery of the cortex, but will also damage the bone medullary area [7]. Not only that, but the growth reduction of blood vessels will also minimise the blood supply in the joint, causing the cortical bone to resorb [8]. These problems need to be resolved as the number of total hip replacements each year is increasing, and therefore a better prosthesis design is needed [9].

In order to fulfil the patient’s requirements and market needs, a survey was conducted and new proposed solutions were suggested in a previous paper. The opinions from the survey were considered, and the suggestions were to develop hip implants by using materials that guarantee adequate biocompatibility, strength and lightweight [10]. Moreover, there are studies on subject animals that result in good osteointegration in the implantation area when lightened and porous hip implants are used [11,12]. In addition, Yang et al. [8] proposed that implants have to be lightweight to provide greater area and improve medullary revascularization. The design could involve manipulating the unique geometries of the implant by applying a lattice structure to reduce hip implant masses by up to 15–17%.

Moulton et al. [13] conducted a study which indicated that the uncemented technique for THA is becoming increasingly accepted and linked with successful outcomes. Previous research has demonstrated that primary THA patients utilising an uncemented tapered femoral component had high survival rates of up to 29 years [14]. Nevertheless, other studies have emphasised the importance of selecting an appropriate size for the uncemented femoral component in order to achieve an optimal stability of the implant during the initial stages [13]. As a result, the current study developed uncemented implants with varying lattice structures utilising commercial geometry.

Based on all of the problems that arise, the necessity of porous and lightweight hip implants is important in order to provide the best possible treatment to patients. By implementing porous and lightweight hip implants during THA operation, it can resolve most of the issues by providing more space for better osteointegration, improve medullary revascularization and improve the blood circulation of the patients [15]. A lightweight implant can be obtained by changing the materials from 316 L stainless steel to titanium alloy (Ti6Al4V), as well as pore designation [16]. However, the topology optimization of the hip implant is still questionable, thus leading to this study, where an analysis was performed on hip implants with different lattice structure that affect the mass of the hip implant [17]. Therefore, this study was conducted to study the biomechanical effects of the commonly used lattice structures of Gyroid and Voronoi on the uncemented hip implant design through finite element analysis. This study is not only meaningful for orthopaedic surgeons to justify their choices of hip implant design, but also could contribute new knowledge in understanding the biomechanical characteristics of porous implants for future studies.

## 2. Materials and Methods

### 2.1. Reconstruction of Femur Bone Model

A computed tomography (CT) dataset of a 27-year-old male weighing 75 kg and standing at 169 cm tall was used to create a three-dimensional (3D) model of the femur bone. The segmentation process was performed in Mimics software version 21.0 (Materialise, Leuven, Belgium) [18,19]. In general, the CT scan data captured the whole human anatomy of the lower-limb region. To obtain the 3D image structure of the femur bone, segmentation was conducted, whereby the threshold was defined for femur bones ranging from 701 HU to 3021 HU to highlight the cortical bone and solely focus on the bone area.

### 2.2. Mesh Convergence Study

**Figure 1:**
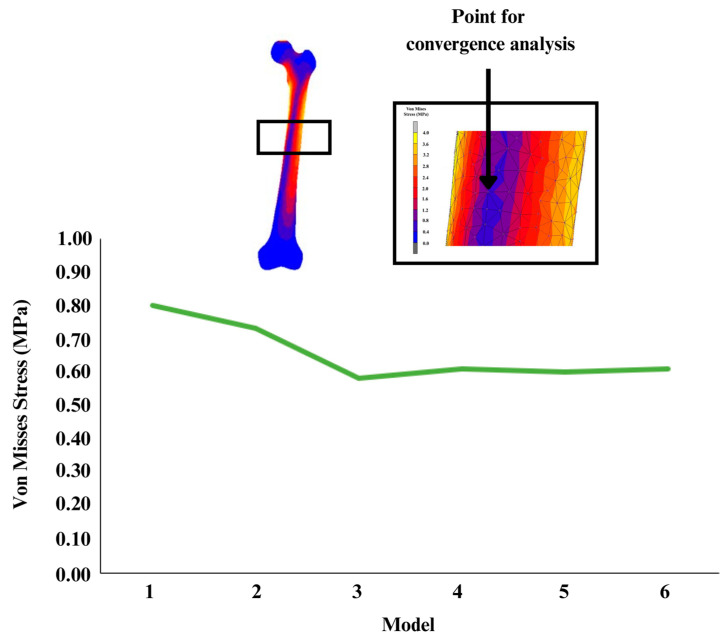
Graph of convergence study.

A convergence analysis was conducted to guarantee the quality of the femur model so that the findings would not be impacted by varying mesh sizes. The 4-node tetrahedral element was used for the bone model, since it has been shown to be more precise than its corresponding node element [20]. Furthermore, the h-refinement strategy was used in this convergence study since it may enhance finite element results by using a finer mesh size [21]. This strategy is known as lowering element length (h) by splitting each existing element into many elements that do not impact the kind of tetrahedral or hexahedral components used [22]. There were six different models refined by using the 3-Matic software version 13.0 (Materialise, Leuven, Belgium), with mesh sizes ranging from 3.5 mm to 6 mm. Table 1 displays the information for each model used in the convergence analysis. For the simulation, the proximal femur was compressed by 375 N (half of the body weight) to replicate a standing leg using MacNeal-Schwendler Corporation (MSC) Marc Mentat (MSC Software Corporation, Munich, Germany) [23]. Based on Figure 1, it was seen that the stress distribution dropped as the number of elements increased until it stayed almost constant by the fourth try, and in order to identify the convergence point, the percentage error between two successive models was computed. Since the discrepancies were less than 5% for model 3 to model 6, the model was considered to have converged at that time [24]. This assumption was supported by the graph shown in Figure 1, which demonstrated that convergence begins at model 3. Hence, model 3 (4.5 mm) was selected as the optimum mesh size for this investigation, which was almost equivalent to previous studies [25]. The majority of prior studies conducted experiments with a mesh size of 4 mm, a difference of 0.5 mm from this study; nevertheless, the mesh size was still within the allowed range because it varies depending on the model employed [25,26].

### 2.3. Porous Hip Implant

**Figure 2:**
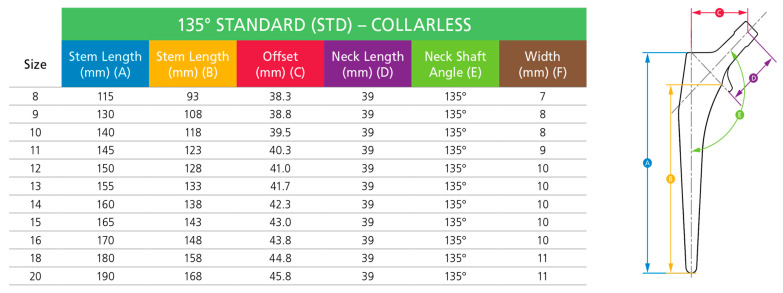
Stem specifications from the CORAIL Hip System [27].

**Figure 3:**
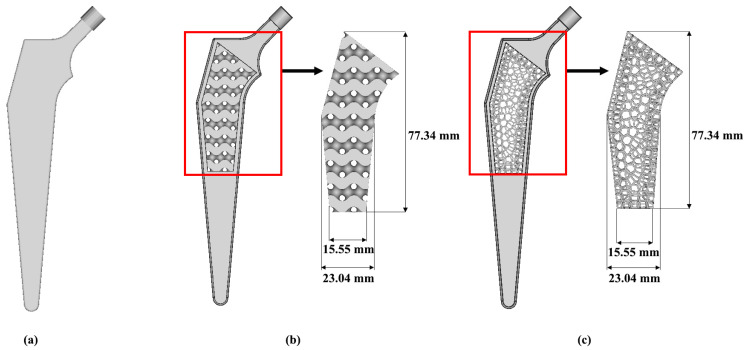
Hip implants model for (**a**) solid, (**b**) Gyroid and (**c**) Voronoi design.

A porous hip implant was constructed by using Computer Aided Design (CAD), SolidWorks version 2020 (Dassault, MA, USA) and nTopology software. The geometry of hip implant was constructed and referred from CORAIL Hip System (Depuy Synthes) [27]. The parts that were considered in the CORAIL Hip system brochure were the neck-shaft angle, long stem length, middle stem length, offset, neck length and width of the hip implant, as illustrated in Figure 2. The design of the implant was established based on a survey administered to expert surgeons and academics working in various universities and hospitals [7]. This led to the selection of a reference geometry with a stem length of 160 mm for the three-dimensional (3D) modelling of the implant [7]. For hip implant topology optimisation, a naturally derived lattice was required to imitate its function. Thus, it was important to ensure that the implants were able to cope with the greater loads associated with body weight and mobility. Therefore, a Voronoi structure was a good selection as it resembles the interior trabecular structure, while Gyroid-like structures can be found at the butterfly wing complex, which appears to have a high endurance to bending loads [28]. A set of holes were created in the mid-region of both the Gyroid and Voronoi designs, following the pattern of lines depicted in Figure 3b,c, respectively. These two structures were applied in the middle part of the hip implant. For the material of hip implant, appropriate mechanical properties were considered. Titanium alloy Ti6Al4V was used for the hip implant, and it is known that its characteristics, including being lightweight, strong and biocompatible, are suitable and commonly used in orthopaedic implants [29].

### 2.4. Finite Element Model

In this investigation, a finite element model was presumed to be isotropic, homogeneous and linear for the femur [19] and hip implant [30]. Tetrahedral elements were applied to all of the implant designs [31]. The Young’s modulus and Poisson ratio of the femur cortical bone were constant for all of the designs at 16.2 GPa and 0.3, respectively [32]. Meanwhile, titanium alloys were selected for this study as the hip implant material, in which the properties were assigned a Young’s modulus of 110 GPa and a Poisson ratio of 0.342 [33]. In general, a femur implanted with a solid hip implant had a total of 1,451,371 elements. Meanwhile, the Gyroid and Voronoi designs consisted of 1,254,474 and 13,777,723 elements, respectively.

### 2.5. Boundary Conditions

**Figure 4:**
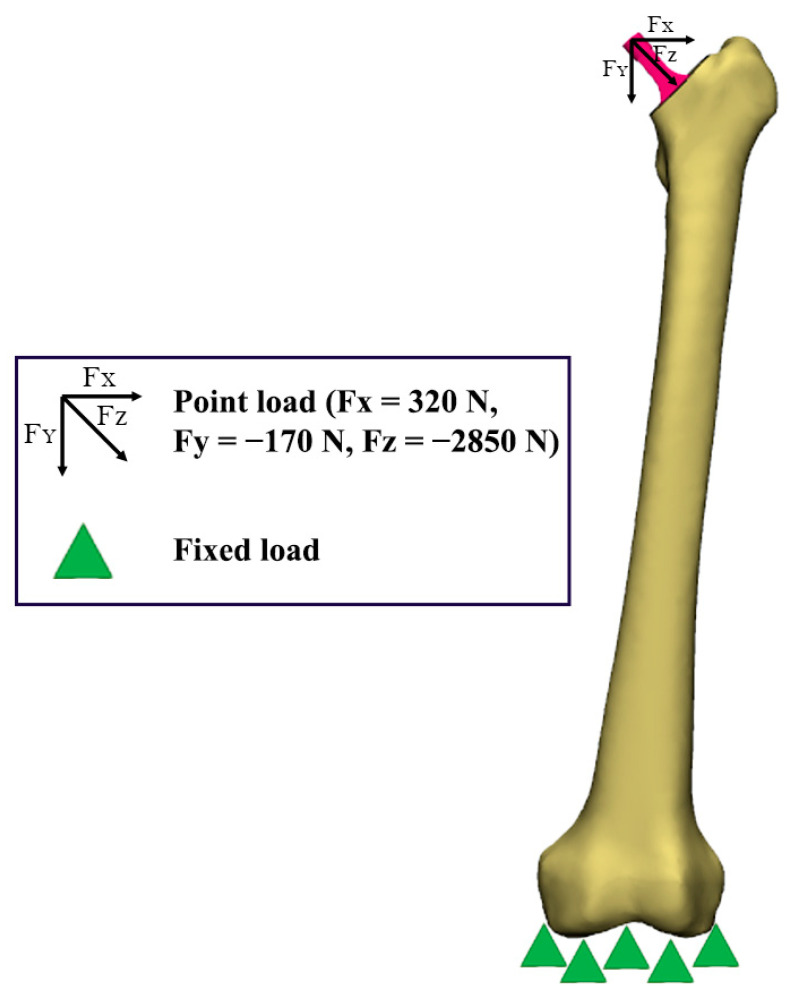
Loading and boundary conditions.

To imitate the stance phase condition, point loads were applied at the x, y and z axis with forces of Fx = 320 N, Fy = −170 N, and Fz = −2850 N, as demonstrated by a previous study [31,32]. To limit the movement of the model, fixed displacement was set at the x, y and z axis, whereby it was fully constrained at the bottom part of the femur [31]. Meanwhile, to promote long-term osteointegration, a bonded contact type was employed for the femur–implant interface during the finite element analysis [34]. The boundary conditions for all three designs were consistently set in this static structural analysis. Figure 4 shows the boundary conditions of the model [31].

### 2.6. Model Validation

**Figure 5:**
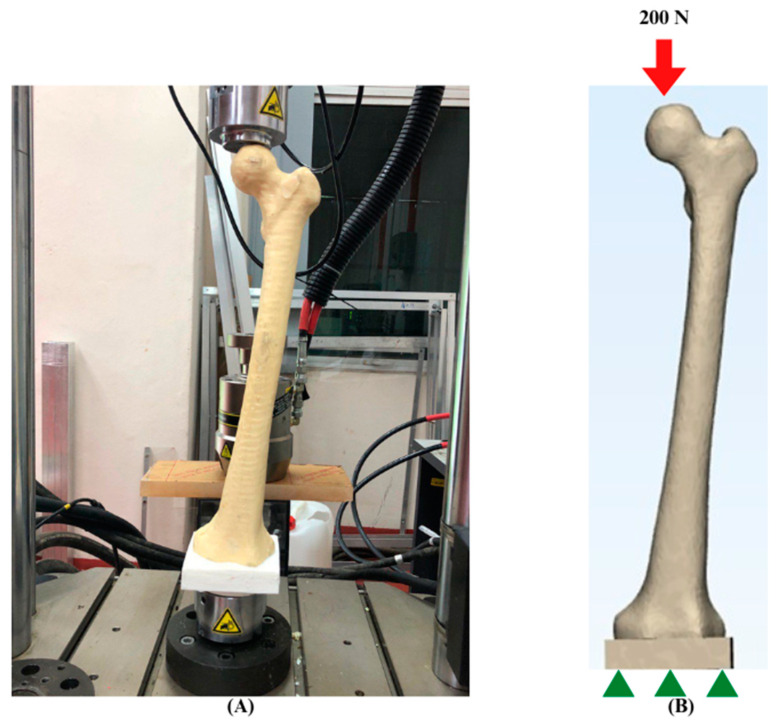
Boundary setup of validation. (**A**) Experimental. (**B**) Simulation.

**Figure 6:**
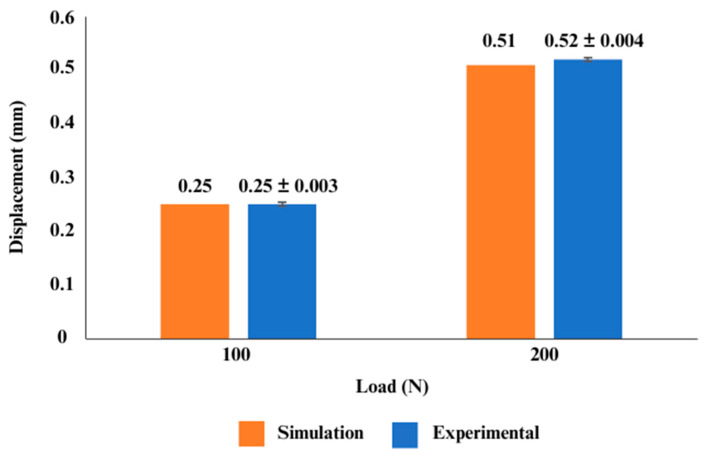
Validation of the femoral bone model. Comparison between experimental and simulation methods.

The finite element (FE) model of the femur was experimentally validated to strengthen the reliability of the analyses. In the context of finite element analysis (FEA), validation is the process of comparing the model predictions to experimental data in order to identify the modelling error [35,36]. The 3D femoral model was generated by using a 3D printer (Zortrax, Olsztyn, Poland), and synthetic bone from polyurethane (PU) was manufactured based on the printed bone sample. As depicted in Figure 5, the synthetic bone was subsequently subjected to 100 N to 200 N axial compression loads, with the distal part of the femur fixed in a custom-made jig utilising an Instron 8874 universal testing machine (UTM) (Instron Ltd., High Wycombe, UK). Meanwhile, the FE femoral model was also subjected to FE analysis, with all properties and boundary conditions being set identically to the experimental configuration. To achieve a similar configuration, 3D scanning was performed by using the Sense^TM^ 3D Scanner (Sense 3D, 3D Devices, Inc., South California, USA) to assure a similar position of the bone and jig. In addition, the bone was assumed to be isotropic, homogeneous, and linearly elastic, with Young’s modulus values and Poisson’s ratio set at 735 MPa and 0.3, respectively, based on the researchers’ previous experiments [37]. Based on the graph shown in Figure 6, displacement was observed during both experiments, and the accuracy of the prediction was determined by the calculation of standard deviations. During a 100 N to 200 N load, it was discovered that the simulation results were superior to the experimental findings. There are a number of reasons that contribute to variations between simulation and experimental values. One of the reasons is the position of the load node in the simulation, which might differ significantly from the area where the experimental load is registered. Secondly, the finite element (FE) model boundary conditions might introduce some inaccuracies due to the compliance of the attachment at the base of the bone [38]. Nevertheless, based on the calculation of the standard deviations, the value of displacements derived from the simulation fell within the allowed range of displacements for the experiment, as illustrated in Figure 6. Therefore, the femur model employed in this investigation was validated by the experimental results.

## 3. Results

### 3.1. Weight of Lightweight Hip Implant

The mass of each design was weighed in nTopology software, and the mass is tabulated in Table 2. The Voronoi hip implant (103.97 g) was the lightest hip implant as compared to the solid (160.68 g) and Gyroid (127.86 g) designs. As a percentage, the Gyroid and Voronoi implants yielded a lighter weight by around 20% and 35%, respectively, as compared to the solid hip implant.

### 3.2. Stress Distribution

**Figure 7:**
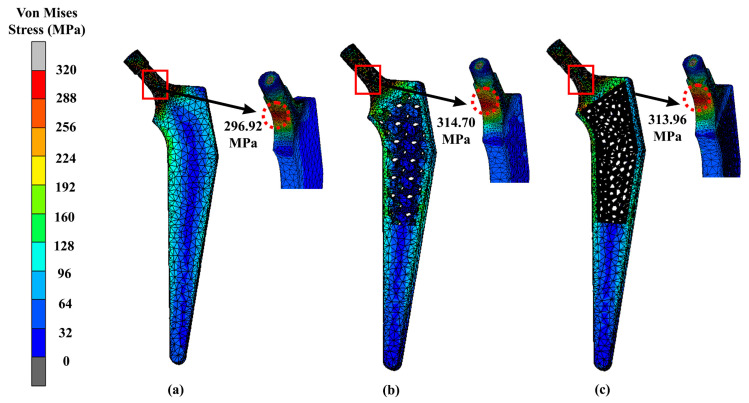
Maximum stress of (**a**) solid, (**b**) Gyroid and (**c**) Voronoi hip implants using titanium alloy.

**Figure 8:**
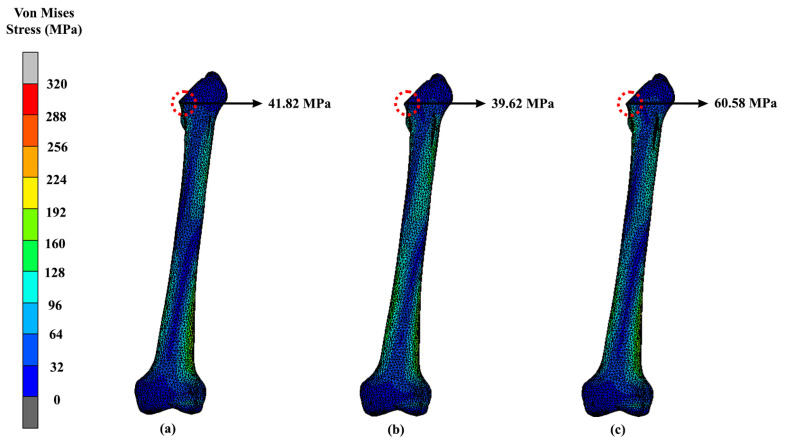
Maximum stress of the femur that attaches to (**a**) solid, (**b**) Gyroid and (**c**) Voronoi hip implants using titanium alloy.

The findings are presented through contour plots in Figure 7 and Figure 8, which show the results of the implant and femoral bone, respectively. According to Table 3, the solid hip implant demonstrated the lowest stress amongst the designs, while the Voronoi implant’s stress values were 5.6% greater than those of the solid implant and only 0.2% higher than those of the Gyroid implant. All designs used titanium alloys which had a Young’s modulus of 110 GPa and a Poisson ratio of 0.3. As shown in Figure 7, the maximum stress was located at the same neck region area of the hip implant for all three designs. The hip implant was attached to the femur bone of the same CT scan patient. The Voronoi design exhibited the highest stress yield for the femur bone (60.58 MPa) as shown in Figure 8. Meanwhile, as shown in Table 4, solid and Gyroid designs had maximum stresses of 41.82 MPa and 39.62 MPa, respectively. As for the femur, the maximum VMS was observed concentrated at the intersectional part of the bisected bone and the results were consistent amongst all three femurs, regardless of the hip implant structures used.

**Figure 9:**
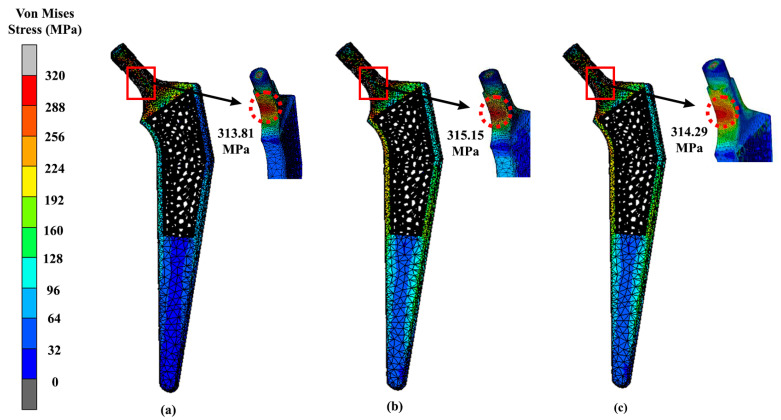
Maximum stress of Voronoi implants using (**a**) magnesium alloy, (**b**) stainless steel and (**c**) cobalt-chromium.

**Figure 10:**
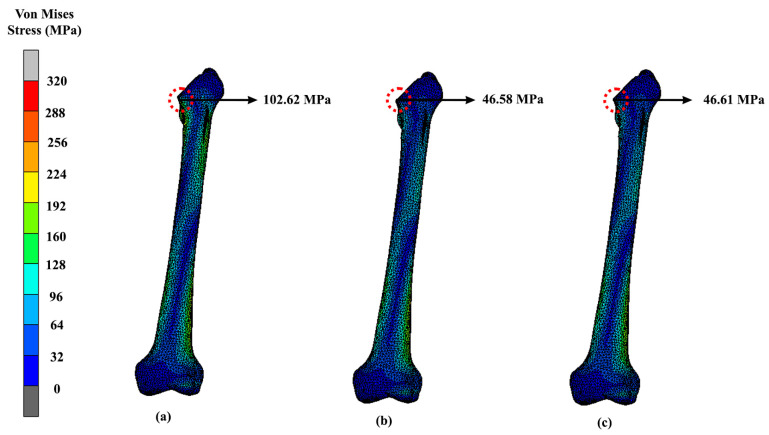
Maximum stress of the femur that attaches to the Voronoi implant using (**a**) magnesium alloy, (**b**) stainless steel and (**c**) cobalt-chromium.

The lightest hip implant, which is the Voronoi design, was also tested with different implant materials other than titanium alloys, such as magnesium alloy, stainless steel and cobalt-chromium. Voronoi implants yielded 314.67 MPa, 315.15 MPa and 314.29 MPa for magnesium alloy, stainless steel and cobalt-chromium, respectively, as shown in Figure 9. When calculating the average difference between the stainless steel and magnesium alloy hip implants, it displayed a 2% difference in terms of their maximum VMS. Meanwhile, the other two materials, which are cobalt-chromium and titanium alloy, did not show a lot of difference in their maximum stress value between them. The difference on average between titanium alloy and cobalt-chromium was only around 0.1%, which indicated not much of a divergence. Meanwhile, as for the femur bone, the maximum VMS was observed concentrated at the intersectional part of the bisected bone, and the results were similar to all three femurs implanted with magnesium alloy, stainless steel and cobalt-chromium materials (Figure 10). Referring to the results, the highest maximum stress at femur was experienced by magnesium alloy hip implants. The Voronoi design yielded 102.62 MPa, 46.61 MPa and 46.58 MPa for magnesium alloy, cobalt-chromium and stainless steel, respectively.

**Figure 11:**
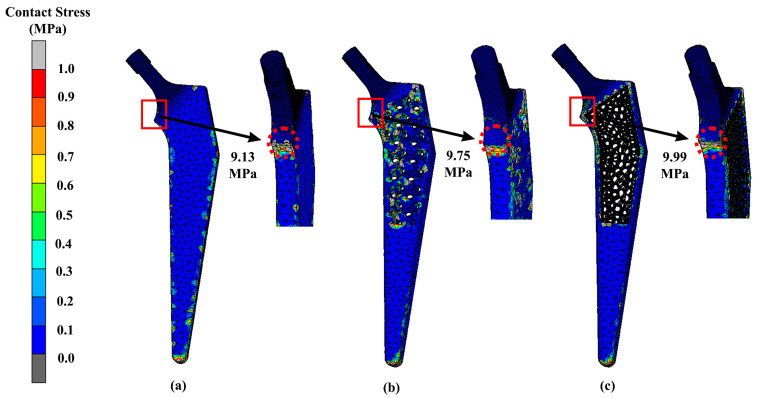
Contact stress of (**a**) solid, (**b**) Gyroid and (**c**) Voronoi hip implants using titanium alloy.

**Figure 12:**
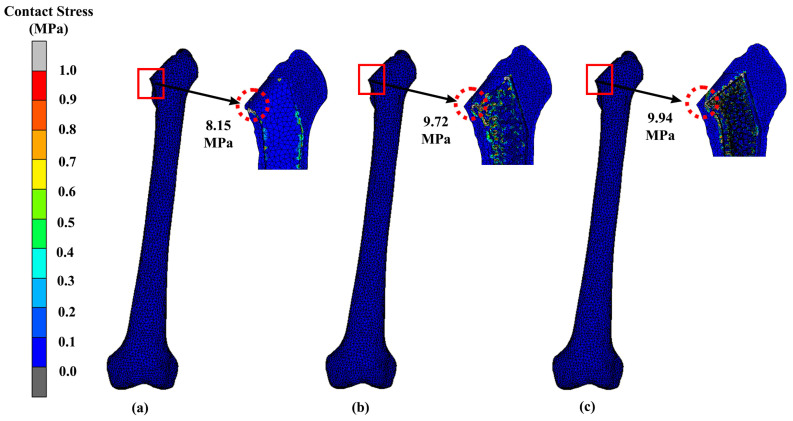
Contact stress of the femur that attaches to a (**a**) solid, (**b**) Gyroid and (**c**) Voronoi hip implant using titanium alloy.

Contact stress is a critical factor in determining the load-bearing capacity of machine components and structures. The results of our analysis revealed that the contact stress distribution was highly dependent on the surface geometry, load distribution, and material properties. The highest contact stresses were observed at the neck region of the implant and bone where the load was concentrated. The implant contact stress magnitudes from our results were 9.13 MPa, 9.75 MPa, and 9.99 MPa for the solid, Gyroid, and Voronoi designs, respectively (Figure 11). For the femur bone, the stress magnitudes were 8.15 MPa, 9.72 MPa, and 9.94 MPa for the solid, Gyroid, and Voronoi designs, respectively (Figure 12).

### 3.3. Displacement

**Figure 13:**
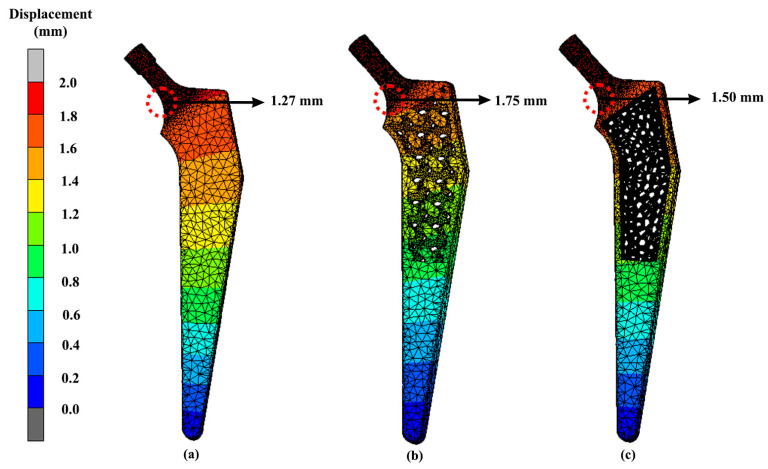
Implant displacement for (**a**) solid (**b**) Gyroid and (**c**) Voronoi hip implants using titanium alloy.

**Figure 14:**
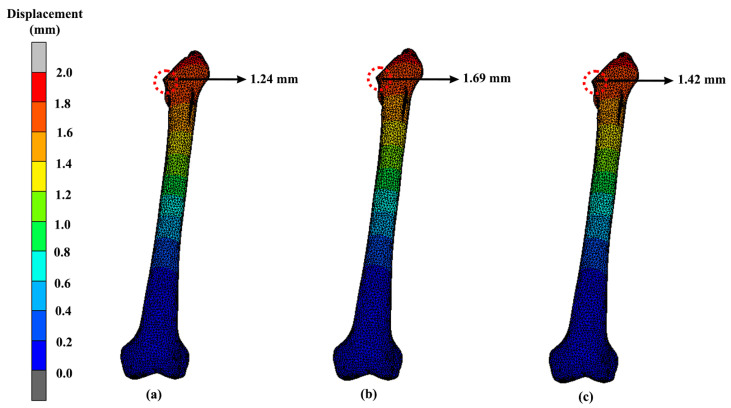
Bone displacement when attached to (**a**) solid (**b**) Gyroid and (**c**) Voronoi hip implants using titanium alloy.

The displacement of the implants can be seen in Figure 13. The highest displacement of the solid, Gyroid and Voronoi models were all located at the same area at the neck with values of 1.27 mm, 1.75 mm and 1.50 mm, respectively. Figure 14 shows the peak displacement that occurred in the femur attached to the implants. The first femur that was attached to the solid implant had 1.24 mm of displacement. Meanwhile, the femur that was attached to the Gyroid and Voronoi implants had displacement values of 1.69 mm and 1.42 mm, respectively.

**Figure 15:**
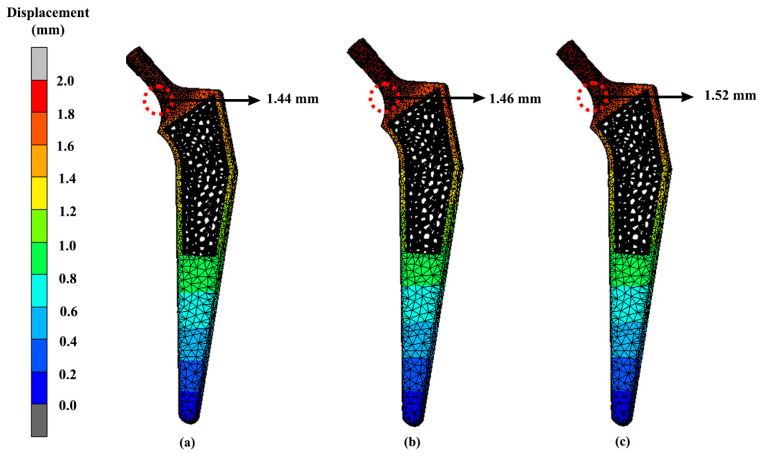
Implant displacement of a Voronoi implant using (**a**) magnesium alloy, (**b**) stainless steel and (**c**) cobalt-chromium.

**Figure 16:**
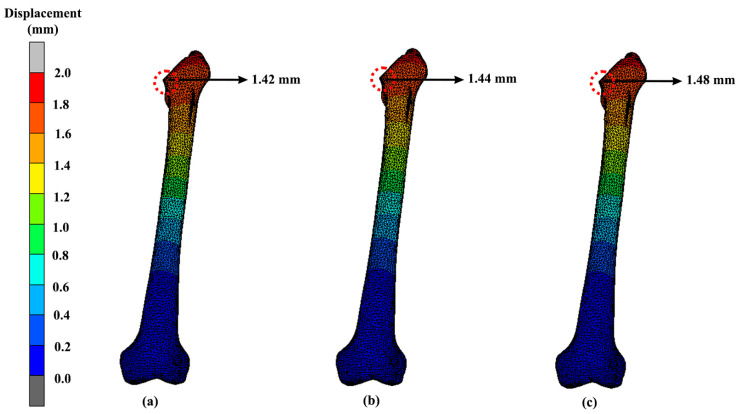
Bone displacement when attached to a Voronoi implant using (**a**) magnesium alloy, (**b**) stainless steel and (**c**) cobalt-chromium.

The displacement of the lightest hip implant design (Voronoi) with different materials such that magnesium alloy, stainless steel and cobalt-chromium is also represented in Figure 15. Referring to the results, the highest displacement was experienced by cobalt-chromium hip implants, while magnesium alloy yielded the lowest displacement. The displacement for magnesium alloy was 1.44 mm, and it was 1.52 mm for cobalt-chromium. Meanwhile, the other two materials, which were stainless steel and titanium alloy, had displacement values of 1.46 mm and 1.50 mm, respectively. Meanwhile, the displacement of the femur was also included in this study, as shown in Figure 16. The displacement pattern observed for the femur bone was similar to its implant. The highest displacement was shown for the femur that was attached to the magnesium alloy hip implant. The displacement values were 1.42 mm, 1.48 mm and 1.44 mm for magnesium alloy, cobalt-chromium and stainless-steel implants, respectively.

## 4. Discussion

The main aim of this study was to design a lightweight hip implant and carry out an investigation of the effect of introducing a lattice structure at the stem of the prosthesis. It also focused on improving the long-term success of hip implants by integrating a porous structure into the stem. This design facilitates bone ingrowth, enhancing the implant’s stability over time [39]. To further ensure success, stress shielding must also be minimised. When designing lightweight hip implants, the topological structures, namely Gyroid and Voronoi, were chosen because of their potential mechanical strength, as demonstrated in previous investigations [18]. In addition, all three implants were weighed by using mass distribution features in nTopology software in order to obtain the lightweighting rates. Referring to the results, there was a significant difference between the solid implants and lightened ones. This is because lattice structures are made up of void constructions of 3D unit cells which are then arranged in a regular pattern, which makes them low in weight as compared to solid structures. In addition, previous research explained that modification of the structural properties of the implant, such as a graded lattice, resulted in a decrease in stiffness [40]. Consequently, these structures help to reduce both bone resorption and interface failure at the same time [41]. Due to decreased stiffness of the lattice structure, several areas of the implant may experience high stress when compared with a solid implant, as demonstrated in Figure 7 and Figure 9 at the neck region.

In addition, according to another study, topology optimisation can help in the mass distribution that led to a lighter hip implant. In comparison with a previous study by Delikanli and Kayacan [7], they found that applying KRZ topology can reduce the mass of hip implants by 15–17% as compared to solid hip implants. Likewise, from the recent results in this study, topology optimisation through Gyroid and Voronoi lattice structures showed a high mass reduction of around 20% and 35%, respectively. The percentage difference of the mass reduction might be caused by the different lattice structures applied in the previous literature and this study. This study also concluded that solid hip implants should have a lower VMS and displacement than lightened implants due to their smaller cross-sectional area and higher stiffness [7]. The results from this study were in accordance with the findings in the previous literature. Referring to the results, the magnitude of VMS and displacement for each of the lightened hip implants appeared higher than solid implants.

High stress can lead to femoral stem fractures, whereas past research has mentioned that the failure of hip implants is caused by several circumstances, including increasing stress in the stem as a result of patients getting heavier, intense activity, or an undersized hip implant [37,42,43]. According to the FEA results (Figure 7), the topology optimisation approach in designing lightened hip implants revealed that hip implants with a Voronoi structure had the highest strength. A Voronoi lattice structure presented the lowest maximum stress value, which was 313.96 MPa, as compared to Gyroid (314.70 MPa). Even so, this stress only existed at a certain location, which meant it did not affect the overall implant’s mechanical behaviour, as other stresses ranged from 10 MPa to 300 MPa. Nevertheless, as expected, the solid implant yielded the least stress compared to the lightened ones. Furthermore, the lightened protheses have a smaller and more complex cross-sectional area, which leads them to encounter substantially higher stresses under the same load [7].

Additionally, materials for hip implants also play an important role in the proper application of prostheses. As noted, all three of these different implants underwent the same simulation and, as expected, the solid implant yielded the least stress and smaller displacements, similarly to in a previous study. However, between the two lattice structures, Voronoi showed promising results as a lightweight hip implant. Based on the findings, magnesium alloy produced the lowest von Mises stress amongst the four materials. Magnesium alloys are known to have a small Young’s modulus of 45 GPa, which is close to the natural bone Young’s modulus of 3 GPa–20 GPa [44]. As a result, the stress shielding caused by the significant mechanical mismatch between the femur and prosthesis can be reduced [45]. However, the yield strength of magnesium alloy was only 85 MPa–190 MPa [46], which was lower than the yielded VMS values. Any deformation caused by stress greater than the yield strength is irreversible, and may increase the risk of metal fracture. Therefore, according to the results, it was revealed that titanium alloy is most preferable material in this study, as it produced the least stress distribution and displacement. Compared to magnesium, titanium alloy is a low-density, high-strength metal that is appropriate for use as an implant because of its high resilience to repeated stresses [47]. It has good mechanical qualities and a high strength-to-weight ratio [48]. In comparison with stainless steel and cobalt-chromium, titanium alloy is less stiff because it has a lower modulus of elasticity, limiting the amount of stress on bone structures [49]. Aside from the implant, the stress distribution can be seen at the intersectional part of the implant and the bisected bone. Figure 10 shows that magnesium alloy yielded the highest stress (102.62 MPa) to the femur bone as compared to cobalt-chromium and stainless steel, which yielded stress values of 46.61 MPa and 46.58 MPa, respectively. Referring to the previous literature, this happened because magnesium is known to have poor mechanical strength and a lower elastic modulus than other materials [34]. However, the low elastic modulus is beneficial to prevent a mismatch between the bone and implant, which can lead to stress shielding. In future studies, it is recommended to improve its strength by alloying the elements implant [50].

As shown in Figure 7, the maximum stress is located at the neck region area of the hip implant for all three designs. The observation was similar to another study, in which the maximum VMS occurred below the neck for the medial part, and as for the lateral side, it happened around one-third of the distance from the neck region [35,51]. Not only that, but the von Mises stress was also distributed throughout the implant until the distal part. The reason for this is because the static system behaves like a cantilever beam, with the bending moment occurring at the fixed boundary condition [28]. Overall, it can be stated that the results were within the safe range, since the magnitude of VMS at each of the implant was lower than the yield strength of Ti6Al4V, which is around 800 MPa–900 MPa [19].

Meanwhile, for the femur bone (Figure 8), the maximum VMS was observed as being concentrated at the intersectional part between the bone and the implant, whereby the results were similar to all three femurs that were implanted with solid, Gyroid and Voronoi structures. The obtained location of VMS was similar to a previous study [52]. Nevertheless, in these analyses, the bones were also considered in a safe range, as the von Mises stress was less than the yielding strength of natural bone at approximately 3 GPa–20 GPa. Overall, the Voronoi structure was most suitable lattice structure found for hip implants. The Voronoi lattice structure produced the lightest implant and yielded the least stress among the lattice structure designs of only 313.96 MPa at the hip implant and 60.58 MPa at the femur. Stress shielding issues might arise when choosing a Voronoi structure as the suitable lattice structure. Using Voronoi structures can help reduce stress shielding and prevent osteolysis [40]. These structures mimic cancellous bone in terms of bionics, mechanical properties, and bone growth, making them suitable for implant design [53]. Additionally, they possess a distinctive combination of a relatively low Young’s modulus and high yield strength, which helps to prevent stress shielding [54]. Nevertheless, in these analyses, the femur bones attached to each of the implants were considered to be in a safe range, as the VMS was less than the yielding strength of cortical bone at 170 MPa [32].

Unfortunately, dislocation of the hip implant can occur after THA, which sometimes leads to a long-term disabling difficulty, especially if there is no proper treatment. Based on the FEA results, the displacements (Figure 13 and Figure 14) seemed to be directly proportional to the value of von Mises stress. Increases in von Mises stress resulted in larger displacements. Again, as expected, the solid hip implant showed a lower displacement as compared to the Voronoi and Gyroid implants. The displacement difference percentage of the Gyroid and Voronoi implants compared with the solid implant was about 32% and 16%, respectively. This proved that solid implants were more rigid than lighter implants, as demonstrated by other studies [28]. However, overly rigid implants can cause stress shielding to occur, which will then contribute to bone loss [31]. Therefore, it is necessary to find an option to reduce stress shielding effects at the bone–implant interface by developing a suitable structure of implant.

As mentioned previously, bone remodelling might occur if local displacements of the implant and bone surpass the allowable deformation of the tissues, which then will lead to the loosening of the hip implant [39]. Furthermore, loosening of the hip prothesis will cause hip pain to the patient and limitation in hip range of motion [53]. Between these two different lattice structure models in this study, the Voronoi structure had a smaller displacement as compared to the Gyroid structure. This demonstrates the Voronoi structure as a better design choice with a lower risk of deformation. Nevertheless, all the designs showed acceptable displacements and can be considered safe, as demonstrated by others [28,31].

The model did not consider the porosity of cancellous bone, as the primary objective of the study was not to examine the bone–implant interface at the microscopic level. Nonetheless, the results were found to be almost identical to the experimental validation of cadavers described in previous papers [55,56]. Therefore, in this study, the FE model geometry consisted of cortical and cancellous bone, and the material characteristics of the bone were simplified and assigned as identical to the prior technique [57], thereby ignoring other key elements that may affect predictions.

While this study focused on testing the design and materials of the implants through compression tests in MacNeal-Schwendler Corporation (MSC) Marc Mentat version 2020 (MSC Software Corporation, Munich, Germany), additional experiments, such as motion, thermal, and fatigue tests, should be conducted to enhance the accuracy and variability of the results. It would also be beneficial to fabricate the design model for carrying out dynamic and fatigue tests to ensure the durability and long-term performance of the hip implants. The results of von Mises stress and displacement presented in this study are therefore valuable for guiding future research and providing insights into the development of lighter hip implants. Both parameters are favourable options to be analysed and have been used by many researchers to check the strength and stability of the implant structure [7,58,59].

## 5. Conclusions

A lightweight hip implant was constructed, whereby the analysis results indicate that a lightweight hip implant had the potential to be implemented as a hip implant for THA. Titanium alloy was used as the material due to its good mechanical strength and natural ability to have low strain rates for ideal specific strength and stiffness. The use of a Voronoi lattice structure in hip implant design has been proven to result in implants which were 35% lighter and experience less stress as compared to other designs. This was due to topology optimisation. Consequently, incorporating a Voronoi structure in hip implant design could enhance their long-term stability and minimise the risk of complications. However, there will always be room for improvement in further study in order to validate all of the results and data.

## Figures and Tables

**Table 1:** Convergence study details of the femur model.

Model	Mesh Size (mm)	Number of Elements	Number of Nodes
1	6.0	56,099	10,491
2	5.5	90,640	16,639
3	5.0	160,794	29,025
4	4.5	220,084	39,058
5	4.0	308,387	53,652
6	3.5	458,143	78,008

**Table 2:** Comparison of mass and percentage differences between solid, Gyroid and Voronoi hip implants.

Design	Mass (g)	Percentage Different Compared with Solid Implant (%)
Solid	160.68	
Gyroid	127.86	20
Voronoi	103.97	35

**Table 3:** Results of different designs on maximum stress and displacement for the implant.

Design	Maximum Stress (MPa)	Displacement (mm)
Solid	296.92	1.27
Gyroid	314.70	1.75
Voronoi	313.96	1.50

**Table 4:** Results of different materials on maximum stress and displacement for the femur.

Design	Maximum Stress (MPa)	Displacement (mm)
Solid	41.82	1.24
Gyroid	39.62	1.69
Voronoi	60.58	1.42

## Data Availability

The data presented in this study are available on request from the corresponding author.
